# Supplementary material for: Voltammetric Determination of the Total Content of the Most Commonly Occurring Estrogens in Water Media
Source: Molecules. 2025 Feb 6;30(3):751. doi: 10.3390/molecules30030751 (PMC11821142; doi:10.3390/molecules30030751)
Supplement: Supplementary file 1 [file molecules-30-00751-s001.zip › molecules-3411417-supplementary.pdf]

Voltammetric Determination of the Total Content of the Most Commonly Occurring Estrogens in Water Media

Jaromíra Chýlková[1], Jan Bartáček[2,*], Natálie Měchová[1], Miloš Sedlák[2] and Jiří Váňa[2]

[1]Institute of Environmental and Chemical Engineering, Faculty of Chemical Technology, University of Pardubice, Studentská 573, Pardubice CZ 532 10, Czech Republic

[2]Institute of Organic Chemistry and Technology, Faculty of Chemical Technology, University of Pardubice, Studentská 573, Pardubice CZ 532 10, Czech Republic.

*Correspondence: jan.bartacek@upce.cz

Supporting information

Table of content

Figure S1. EE2 voltammograms during oxidation (pH 2.03)
Figure S2. EE2 voltammograms during oxidation (pH 4.03)
Figure S3. EE2 voltammograms during oxidation (pH 6.06)
Figure S4. EE2 voltammograms during oxidation (pH 9.04)
Figure S5. EE2 voltammograms during oxidation (pH 10.00)
Figure S6. EE2 voltammograms at low concentration range (pH 9.04)
Figure S7. E3 voltammograms during oxidation (1.06–8.48 μmol/L)
Figure S8. E3 voltammograms at low concentration range (0.53–2.66 μmol/L)
Figure S9. E1 voltammograms during oxidation (13.32–91.41 μmol/L)
Figure S10. E1 voltammograms at low concentration range (0.95–4.76 μmol/L)
Figure S11. E2 voltammograms during oxidation (19.12–131.25 μmol/L)
Figure S12. E2 voltammograms at low concentration range (1.92–9.59 μmol/L)

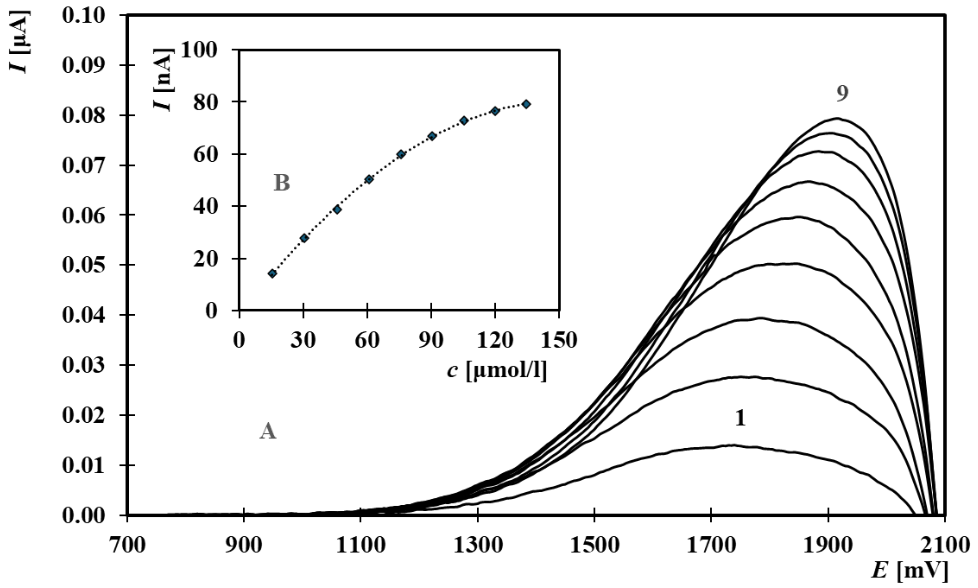

Figure S1. Anodic oxidation curves of EE2 in BR buffer (pH 2.03) with 33% acetonitrile after background subtraction: A - Individual curves (1: 15.35 μmol/L, 9: 134.55 μmol/L), B - Current vs. concentration dependence (15.35–134.55 μmol/L); Parameters: initial potential +700 mV, final potential +2100 mV, polarization rate 25 mV/s, pulse amplitude 30 mV, pulse duration 60 ms.

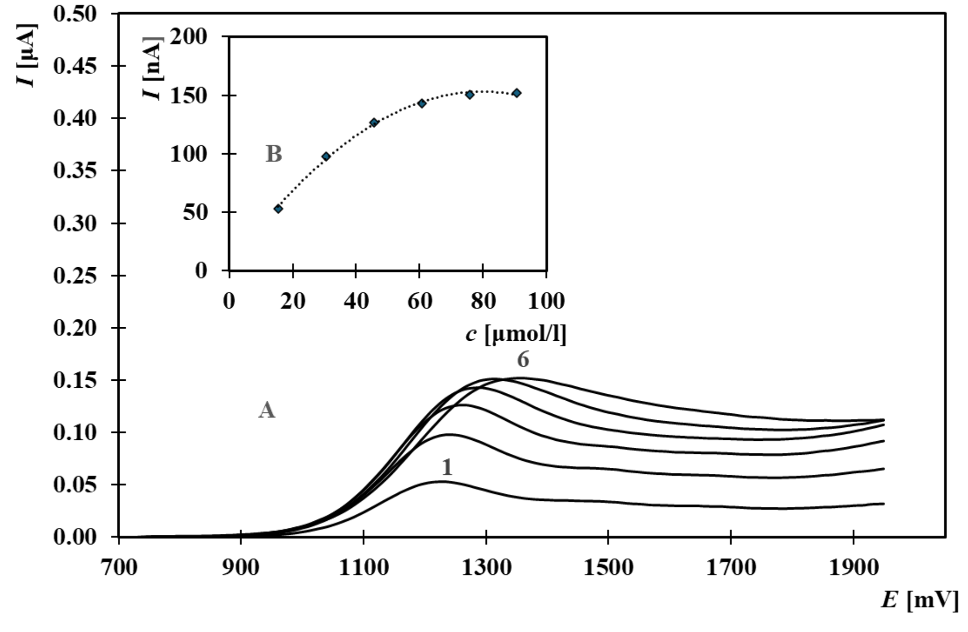

Figure S2. Anodic oxidation curves of EE2 in BR buffer (pH 4.03) with 33% acetonitrile after background subtraction: A - Curves after background subtraction, B - Current vs. concentration dependence (15.35–90.58 μmol/L); Individual curves: 1 - 15.35 μmol/L, 6 - 90.58 μmol/L; Parameters: initial potential +700 mV, final potential +2000 mV, polarization rate 25 mV/s, pulse amplitude 30 mV, pulse duration 60 ms.

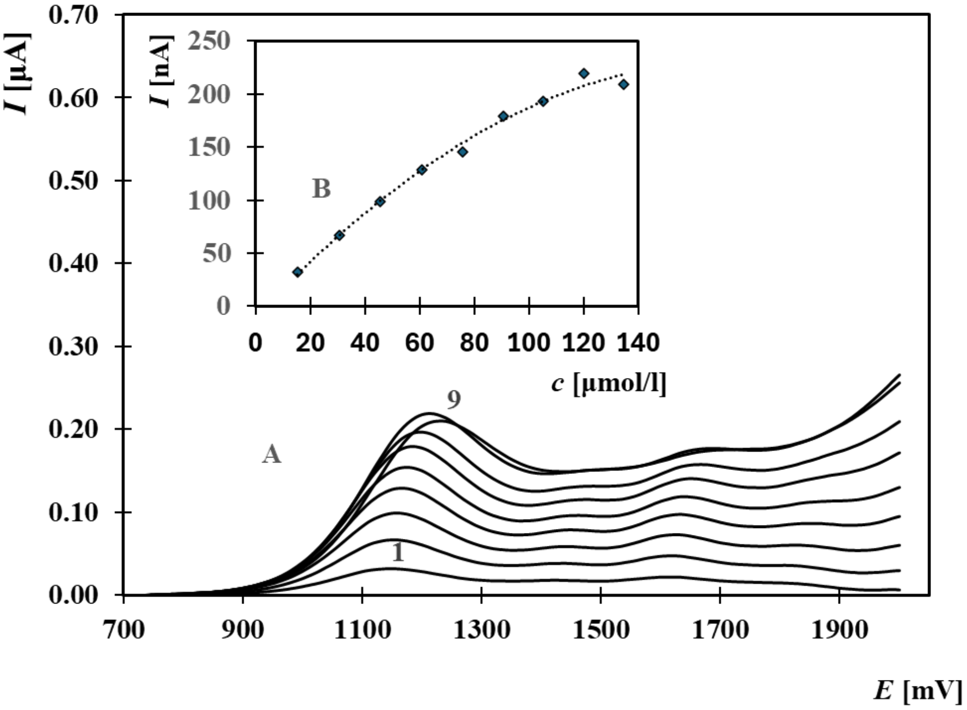

Figure S3. Anodic oxidation curves of EE2 in BR buffer (pH 6.06) with 33% acetonitrile after background subtraction: A - Individual curves (1: 15.35 µmol/L, 9: 134.55 µmol/L), B - Current vs. concentration dependence (15.35–134.55 µmol/L); Parameters: initial potential +700 mV, final potential +2000 mV, polarization rate 25 mV/s, pulse amplitude 30 mV, pulse duration 60 ms.

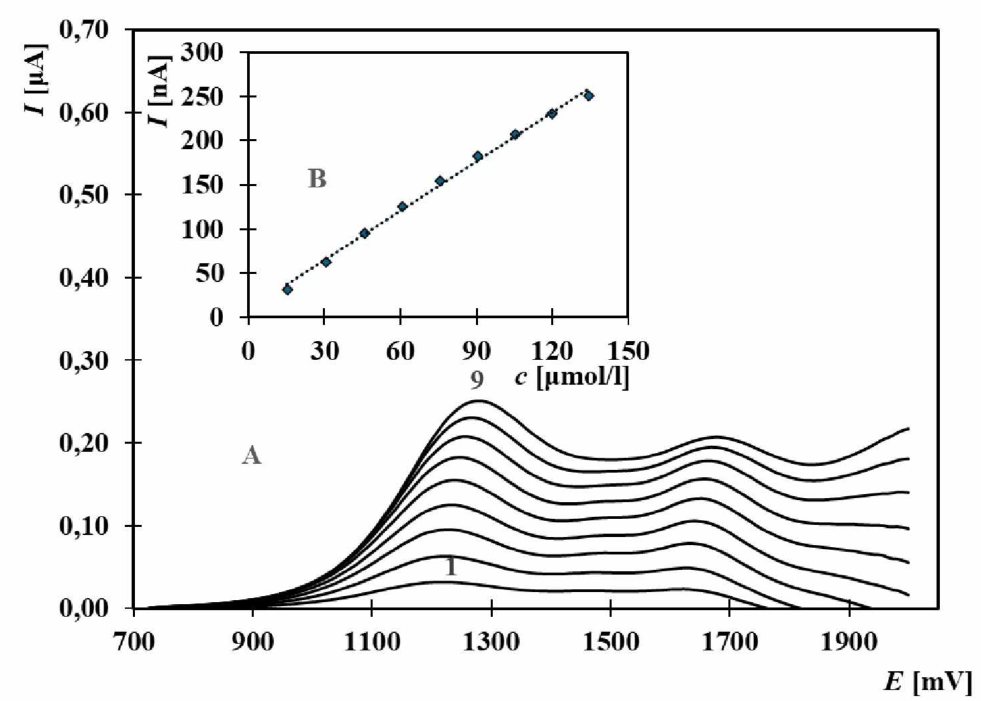

Figure S4. Anodic oxidation curves of EE2 in BR buffer (pH 9.04) with 33% acetonitrile after background subtraction: A - Individual curves (1: 15.35 µmol/L, 9: 134.55 µmol/L), B - Current vs. concentration dependence (15.35–134.55 µmol/L); Parameters: initial potential +700 mV, final potential +2000 mV, polarization rate 25 mV/s, pulse amplitude 30 mV, pulse duration 60 ms.

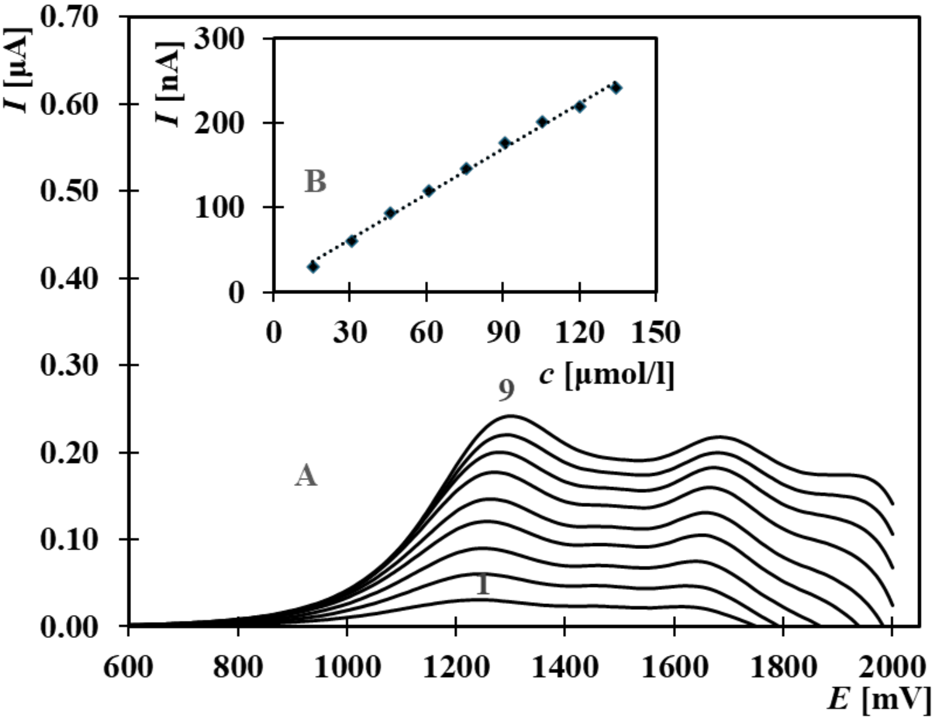

Figure S5. Anodic oxidation curves of EE2 in BR buffer (pH 10.00) with 33% acetonitrile after background subtraction: A - Individual curves (1: 15.35 µmol/L, 9: 134.55 µmol/L), B - Current vs. concentration dependence (15.35–134.55 µmol/L); Parameters: initial potential +700 mV, final potential +2000 mV, polarization rate 25 mV/s, pulse amplitude 30 mV, pulse duration 60 ms.

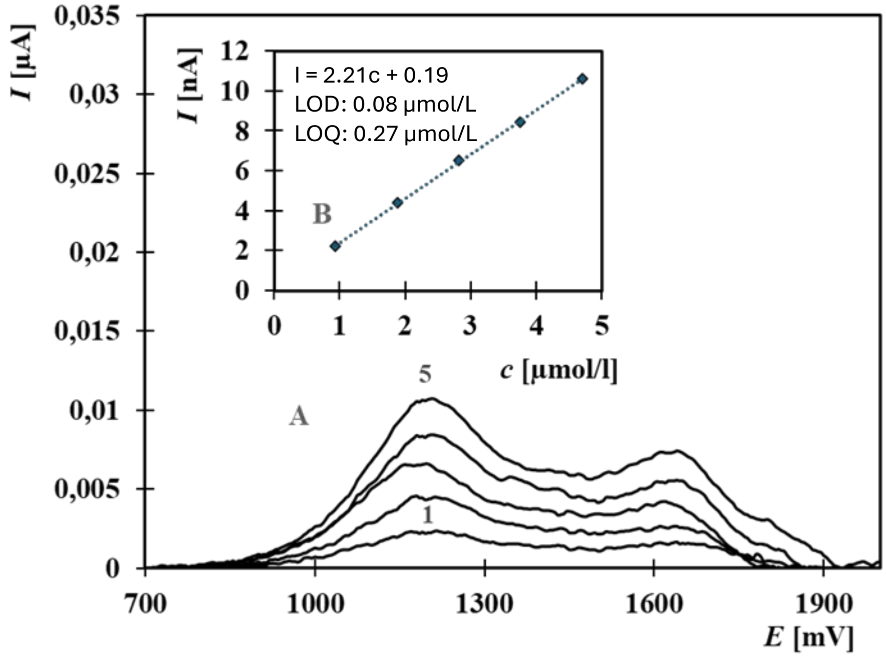

Figure S6. Anodic oxidation curves of EE2 in BR buffer (pH 9.04) with 33% acetonitrile after background subtraction: A – Individual curves (1: 0.94 µmol/L, 5: 4.7 µmol/L), B – Current vs. concentration dependence (0.94–4.7 µmol/L). Parameters: initial potential +700 mV, final potential +2000 mV, polarization rate 25 mV/s, pulse amplitude 30 mV, pulse duration 60 ms.

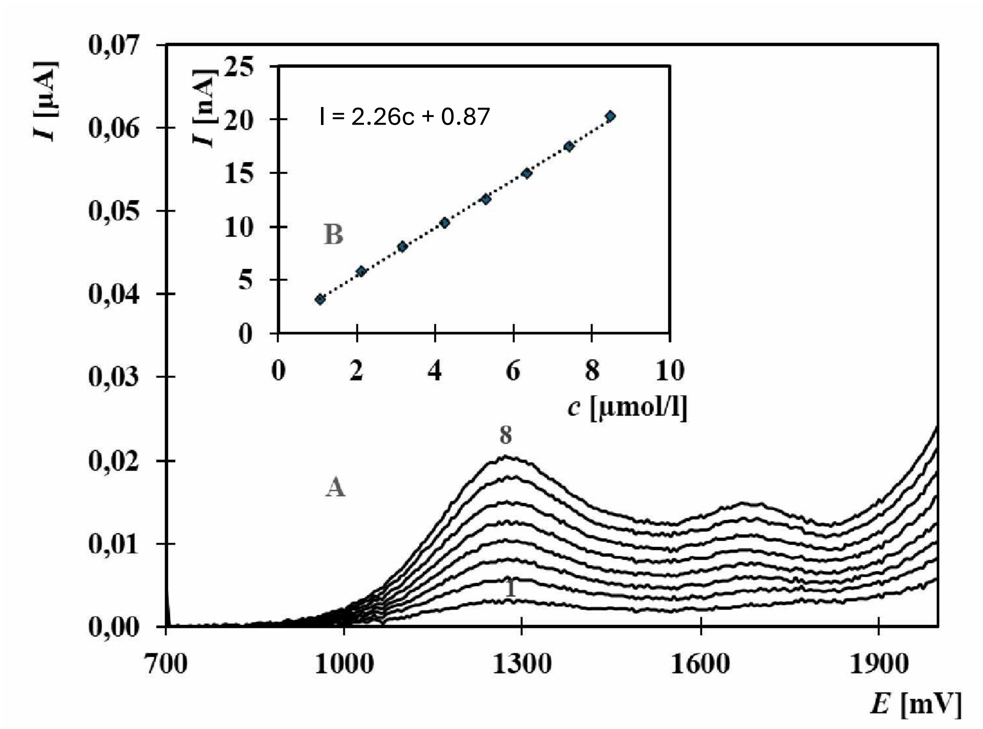

Figure S7. Anodic oxidation curves of E3 in the concentration range 1.06–8.48 µmol/L: A - Curves after background subtraction, B - Current vs. concentration dependence (1.06–8.48 µmol/L); Individual curves: 1 - 1.06 µmol/L, 8 - 8.48 µmol/L; Supporting electrolyte: BR buffer (pH 9.04) with 33% acetonitrile; Parameters: initial potential +700 mV, final potential +2000 mV, polarization rate 25 mV/s, pulse amplitude 30 mV, pulse duration 60 ms.

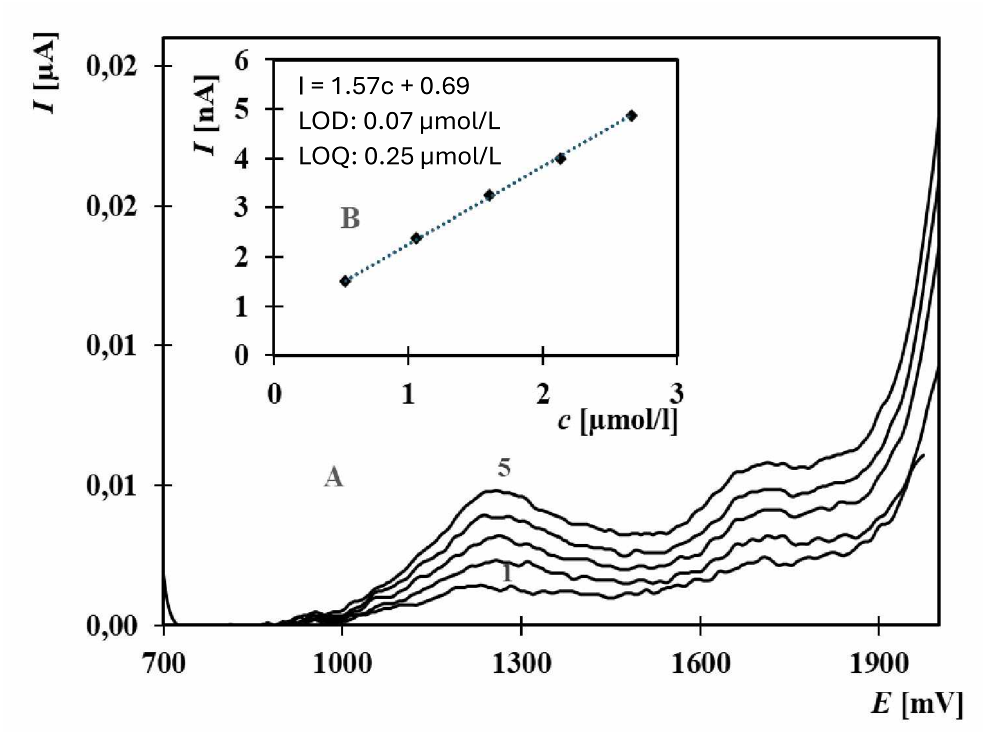

Figure S8. Anodic oxidation curves of E3 in the concentration range 0.53–2.66 µmol/L: A - Curves after background subtraction, B - Current vs. concentration dependence (0.53–2.66 µmol/L); Individual curves: 1 - 0.53 µmol/L, 5 - 2.66 µmol/L; Supporting electrolyte: BR buffer (pH 9.04) with 33% acetonitrile; Parameters: initial potential +700 mV, final potential +2000 mV, polarization rate 25 mV/s, pulse amplitude 30 mV, pulse duration 60 ms.

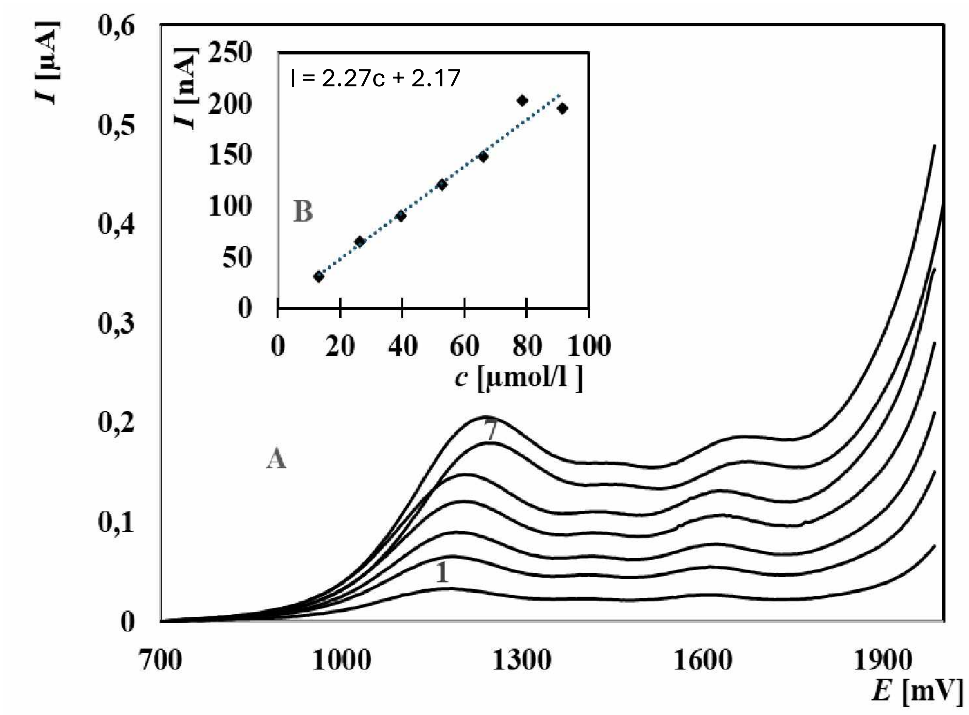

Figure S9. Anodic oxidation curves of E1 in the concentration range 13.32–91.41 µmol/L: A - Curves after background subtraction, B - Current vs. concentration dependence (13.32–91.41 µmol/L); Individual curves: 1 - 13.32 µmol/L, 7 - 91.41 µmol/L; Supporting electrolyte: BR buffer (pH 9.04) with 33% acetonitrile; Parameters: initial potential +700 mV, final potential +2000 mV, polarization rate 25 mV/s, pulse amplitude 30 mV, pulse duration 60 ms.

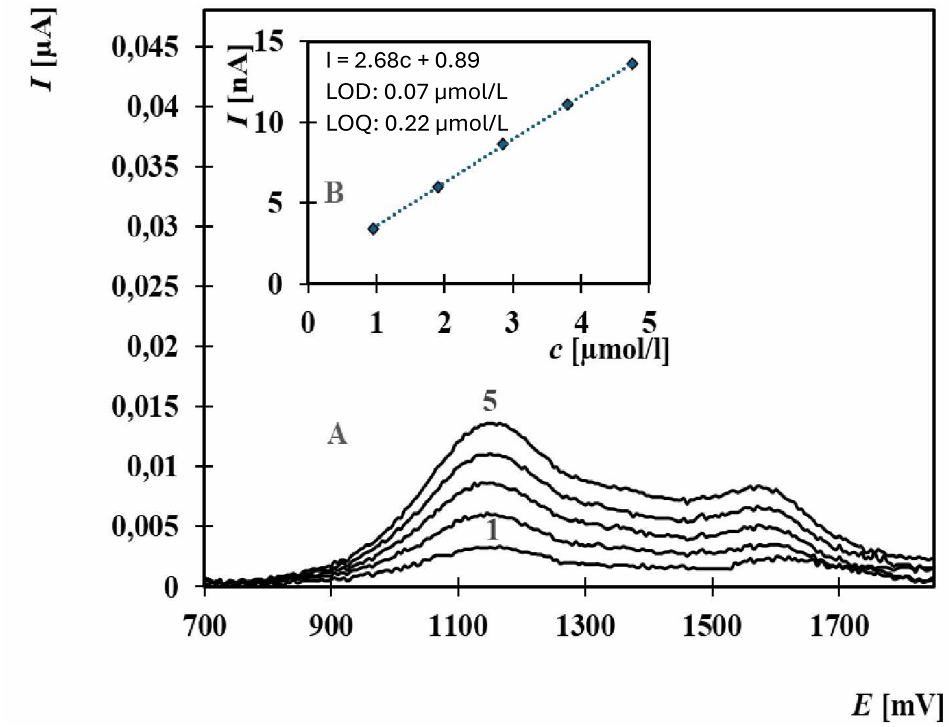

Figure S10. Anodic oxidation curves of E1 in the concentration range 0.95–4.76 µmol/L: A - Curves after background subtraction, B - Current vs. concentration dependence (0.95–4.76 µmol/L); Individual curves: 1 - 0.95 µmol/L, 5 - 4.76 µmol/L; Supporting electrolyte: BR buffer (pH 9.04) with 33% acetonitrile; Parameters: initial potential +700 mV, final potential +2000 mV, polarization rate 25 mV/s, pulse amplitude 30 mV, pulse duration 60 ms.

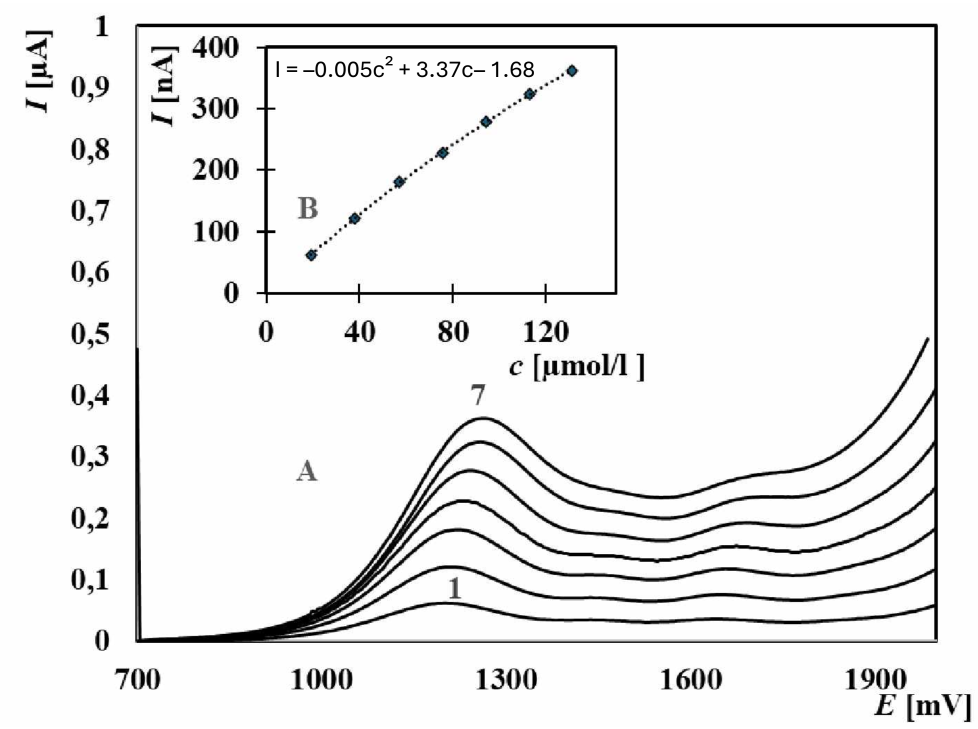

Figure S11. Anodic oxidation curves of E2 in the concentration range 19.12–131.25 μmol/L after background subtraction: A - Curves after background subtraction, B - Current vs. concentration dependence (19.12–131.25 μmol/L); Individual curves: 1 - 19.12 μmol/L, 7 - 131.25 μmol/L; Supporting electrolyte: BR buffer (pH 9.04) with 33% acetonitrile; Parameters: initial potential +700 mV, final potential +2000 mV, polarization rate 25 mV/s, pulse amplitude 30 mV, pulse duration 60 ms.

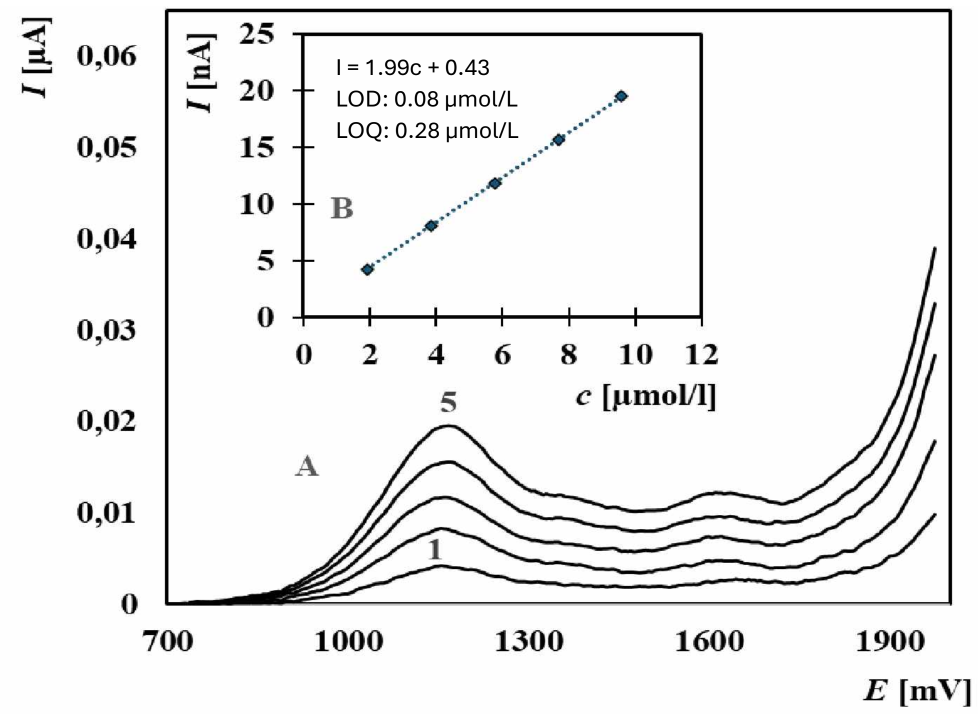

Figure S12. Anodic oxidation curves of E2 in the concentration range 1.92–9.59 μmol/L: A - Curves after background subtraction, B - Current vs. concentration dependence (1.92–9.59 μmol/L); Individual curves: 1 - 1.92 μmol/L, 5 - 9.59 μmol/L; Supporting electrolyte: BR buffer (pH 9.04) with 33% acetonitrile; Parameters: initial potential +700 mV, final potential +2000 mV, polarization rate 25 mV/s, pulse amplitude 30 mV, pulse duration 60 ms.
